# Inflammation and Kidney Injury in Diabetic African American Men

**DOI:** 10.1155/2019/5359635

**Published:** 2019-02-05

**Authors:** Lei Cao, Ava Boston, Olugbemiga Jegede, Heather A. Newman, Scott H. Harrison, Robert H. Newman, Elimelda Moige Ongeri

**Affiliations:** ^1^Department of Biology, North Carolina A&T State University, Greensboro, NC 27411, USA; ^2^Cone Health Community Health and Wellness Center, Greensboro, NC 27401, USA

## Abstract

African Americans are disproportionately burdened by diabetic kidney disease (DKD). However, little is known about the cellular and molecular mechanisms underlying the onset and progression of DKD in this population. The goal of the current study was to determine the association between specific inflammation markers and kidney injury in diabetic African American men. To this end, we recruited diabetic patients either with (*n* = 20) or without (*n* = 87) diagnosed kidney disease along with age-matched nondiabetic controls (*n* = 81). Urinary albumin-to-creatinine ratios (UACRs) and estimated glomerular filtration rates (eGFR) were used for biochemical assessment of kidney function. We then measured plasma and urinary levels of seven inflammatory markers, including adiponectin, C-reactive protein (CRP), tumor necrosis factor-*α* (TNF-*α*), TNF receptor 1 (TNFR1), TNF receptor 2 (TNFR2), interleukin-6 (IL-6), and intercellular cell adhesion molecule-1 (ICAM-1). Plasma levels of TNF-*α*, TNFR1, and TNFR2 were significantly higher in diabetics with macroalbuminuria compared to nondiabetic controls and diabetics with normoalbuminuria or microalbuminuria. Likewise, urinary levels of ICAM-1 were higher in diabetics with macroalbuminuria compared to the other groups. Indeed, urinary ICAM-1, plasma TNF-*α*, and adiponectin had moderate positive correlations with UACR while plasma TNFR1 and TNFR2 levels were strongly correlated with kidney injury, indicated by multiple biomarkers of kidney injury. In contrast, though plasma CRP was elevated in diabetic subjects relative to nondiabetic controls, its levels did not correlate with kidney injury. Together, these data suggest that inflammation, particularly that mediated by the TNF-*α*/NF-*κ*B signaling axis, may play a role in the pathogenesis of DKD in African American men.

## 1. Introduction

The prevalence of diabetes mellitus has reached epidemic proportions in the United States and worldwide. This is largely driven by the obesity epidemic, the primary cause of type 2 diabetes (T2D). The most recent data from the U.S. Center for Disease Control (CDC) suggest that 40% of adults and 18% of youth in the U.S. are obese [1]. In the US, the prevalence of diabetes mellitus (DM) is disproportionately higher in minority ethnic populations, such as African Americans and Hispanics, than in Caucasians [2, 3]. A common complication of diabetes is chronic kidney disease (CKD). Diabetic kidney disease (DKD), which is a leading cause of CKD and end-stage renal disease (ESRD), is estimated to affect nearly 40 percent of all diabetic patients [4]. Treatment of ESRD costs tens of billions of dollars each year. In humans, DKD is characterized by albuminuria [5], renal hypertrophy [6], excess accumulation of extracellular matrix (ECM) proteins within the glomerulus and interstitium [7–10], and a progressively declining glomerular filtration rate (GFR) [11]. The first clinical sign of DKD is microalbuminuria, defined by urinary albumin secretion greater than 30 mg/day or 20 *μ*g/min [12]. Without interventions, 20–40% of patients with T2D and microalbuminuria progress to overt nephropathy, defined by urinary albumin secretion exceeding 300 mg/day or 200 *μ*g/min [12]. The pathogenesis of DKD is not fully understood, but both genetic and environmental factors appear to be involved [13].

Inflammation and subsequent fibrosis have been shown to play a role in the progression of acute kidney injury (AKI) and CKD [13–17]. This is evidenced by glomerular and tubulointerstitial inflammation, accumulation of ECM proteins, and glomerulosclerosis which are characteristic of DKD [18]. Proinflammatory cytokines involved in the pathogenesis of DKD include interleukin-1 (IL-1), IL-6, and IL-18 [13]. Interaction between IL-6 and transforming growth factor *β*1 (TGF-*β*1) has been shown to have a synergistic effect that enhances fibrosis in the kidney [19]. Likewise, elevated serum levels of IL-18 are associated with the progression of DKD [17, 20, 21]. Like macrophages and resident dendritic cells, renal epithelial cells are capable of producing several proinflammatory cytokines (e.g., tumor necrosis factor-*α* (TNF-*α*), IL-6, and TGF-*β*1) as well as chemotactic cytokines (e.g., chemokine (C-C motif) ligand 5 (CCL5/RANTES), monocyte chemoattractant protein-1 (MCP-1), epithelial-derived neutrophil-activating peptide 78 (ENA-78), chemokine (C-X-C motif) ligand 1 (CXCL1), and IL-8 [22–24]). Consistently, previous studies have demonstrated a rise in urinary and renal interstitial TNF-*α* prior to the increase in albuminuria in diabetic rats [25]. Similarly, in mice, TNF-*α* produced by monocytes/macrophages was shown to play a role in diabetic renal injury [26]. Likewise, TNF-*α* depletion was associated with decreases in the levels of the TNF receptors, TNFR1 and TNFR2, suggesting that TNF-*α* may regulate TNFR expression via a positive feedback mechanism [26]. Interestingly, TNF-*α* and adhesion molecules, such as intercellular adhesion molecule-1 (ICAM-1), have been shown to participate in cardinal pathogenetic mechanisms of DKD [13]. A similar role for ICAM-1 in promoting DKD has been documented in mice with T2D [27]. Levels of other inflammation markers have also varied based on the degree of diabetic kidney injury. For instance, both serum IL-6 and C-reactive protein (CRP) positively correlated with urinary albumin excretion in subjects with T2D [28]. Likewise, the plasma levels of adiponectin, an adipose-derived anti-inflammatory protein, decreased in diabetic subjects compared to nondiabetic controls and increased with the progression of kidney injury [29, 30].

Compared to Caucasian Americans, African Americans are at higher risk for developing DKD [31]. Though the cellular and molecular mechanisms underlying the disparities in DKD are not fully understood, there is evidence that inflammatory pathways may play a role. Ethnic differences in inflammation have been observed in other studies. For instance, a 9-year longitudinal study showed that low-grade inflammation predicts the incidence of T2D in Caucasian Americans but not in African Americans [32]. Likewise, in a multiethnic population-based study, significant race and gender differences in the distribution of plasma CRP levels were reported, with women and African American subjects having higher CRP levels than men and Caucasian subjects, respectively [33]. Meanwhile, adiponectin levels in nonobese Caucasian women were higher than those in nonobese African American women [34]. African American children also had lower adiponectin levels than Caucasian children [35]. Finally, factors such as body mass index (BMI) and insulin correlated significantly with adiponectin levels [34]. Interestingly, this correlation was only observed in Caucasian women, but not in African American women [34]. The objective of this study was to evaluate the association between inflammation markers and kidney injury in diabetic African American men, a patient population that is consistently underrepresented in biomedical studies and in analysis of biological samples for development of biomarkers for DKD.

## 2. Materials and Methods

### 2.1. Recruitment of Participants

This study was part of the Minority Men's Health Initiative (MMHI) and was approved by the Institutional Review Boards (IRB) for North Carolina Agricultural and Technology State University and Cone Health. Diabetic African American men 18 to 65 years of age were recruited through the Cone Health Community Health and Wellness Center in Greensboro, NC. Age-matched nondiabetic controls were recruited through local faith-based communities and their diabetic status confirmed via fasting blood glucose assays. Written consent was obtained from all participants before administering questionnaires about personal and family medical history and collecting biological samples. Anthropometric parameters, such as height, weight, waist circumference (WC), and blood pressure, were also determined. For the diabetic patients, medical history was confirmed based on their medical records. Patients with chronic disease conditions not associated with diabetes (e.g., HIV and chronic obstructive pulmonary disorder) and diabetic patients on dialysis were excluded. We recruited a total of 188 participants from three groups: diabetics with no known kidney disease (*n* = 87), diabetics with diagnosed kidney disease (*n* = 20), and age-matched nondiabetic controls (*n* = 81). All participant information was secured using the online clinical data management system, REDCap [36] and deidentified prior to analysis.

### 2.2. Collection and Processing of Biological Samples

Fasting blood and urine samples were obtained from study participants. Blood samples were collected by trained phlebotomists using vacutainers coated with heparin to prevent coagulation (Becton-Dickinson, Sparks, MD). The blood samples were held on ice for an average of 1 hour before being processed to obtain plasma. To obtain plasma, whole blood samples were centrifuged at 2,750 × g for 15 min using a refrigerated Allegra X-14R centrifuge (Beckman Coulter, Brea, CA). The plasma samples (supernatant) were then aliquoted and stored at -80°C. Urine samples were collected into sterile cups and held on ice for an average of 1 hour prior to processing. Collected urine samples were aliquoted and stored at -80°C prior to analysis.

### 2.3. Biochemical Assessment of Kidney Injury

To evaluate kidney function, assays for urinary albumin and creatinine were performed. Creatinine assays utilized a colorimetric assay kit (Diazyme Laboratories, Poway, CA). Albumin levels were determined using enzyme-linked immunosorbent assay (ELISA) kits (Exocell, Philadelphia, PA). Standard curves for albumin, which were generated using a log-log plot, were used to estimate the albumin levels. The urinary albumin-to-creatinine ratios (UACRs) were determined by normalizing the urinary albumin levels to the creatinine levels in the same sample. UACR ≤ 30 mg/g was considered normoalbuminuria, 30 ≤ UACR ≤ 300 mg/g was considered microalbuminuria, and 300 mg/g ≤ UACR was considered macroalbuminuria. Similarly, estimated glomerular filtration rates (eGFR) were determined using the chronic kidney disease-epidemiology collaboration (CKD-EPI) creatinine-cystatin equation described by Inker et al. [37]. Plasma creatinine levels were measured using a colorimetric assay kit (Diazyme Laboratories) while cystatin C levels were determined by ELISA (R&D Systems, Minneapolis, MN). Standard curves for cystatin C were generated using four parameter logistic (4-PL) regression analysis and used to estimate cystatin C levels. Finally, the concentrations of the kidney injury markers, kidney injury molecule-1 (KIM-1) and neutrophil gelatinase-associated lipocalin (NGAL), were determined using ELISA (R&D Systems) according to the manufacturer's instructions. Standard curves were generated using 4-PL regression analysis. Urinary NGAL levels were normalized to urinary creatinine concentrations. DKD is a heterogeneous disease that includes glomerular and interstitial fibrosis and arteriosclerosis phenotypes. For the current study, diagnosed DKD was determined from patient records based on albuminuria and impaired eGFR (<60 mL/min/1.73 m^2^). For patients with undiagnosed DKD, assessments were based on UACR and eGFR, as well as assays for several recently developed biomarkers of kidney injury [38].

### 2.4. Assays for Biomarkers of Inflammation

The plasma and urinary levels of seven markers of inflammation, namely, adiponectin, CRP, IL-6, TNF-*α*, TNFR1, TNFR2, and ICAM-1, were determined using ELISA (R&D Systems, Minneapolis, MN). For these analyses, samples obtained from a subset of the participants in each group were randomly selected for analysis. Each sample was analyzed for all of the inflammation biomarkers indicated above (as well as for UACR, eGFR, and the other kidney injury markers). In total, 64 diabetics (57 without diagnosed DKD and 7 with diagnosed DKD) and 15 age-matched nondiabetic controls were analyzed. All assays were performed according to the manufacturer's instructions. Standard curves were generated using 4-PL regression analysis. The urinary levels of ICAM-1 were normalized to the urine creatinine levels.

### 2.5. Statistical Analysis

One-way ANOVA was used to test the difference among all groups, followed by Tukey's honest significant difference test. The correlation was calculated using Pearson correlation coefficient analysis. The UACR, KIM-1, and NGAL data were log-transformed and subjected to correlation analysis. All statistical analyses were conducted using GraphPad Prism 7.0 (GraphPad Software, San Diego, CA). Data are represented as mean ± SEM. A *p* value ≤ 0.05 was considered statistically significant.

## 3. Results

### 3.1. Clinical Characteristics of Nondiabetic and Diabetic Subjects

**Figure 1:**
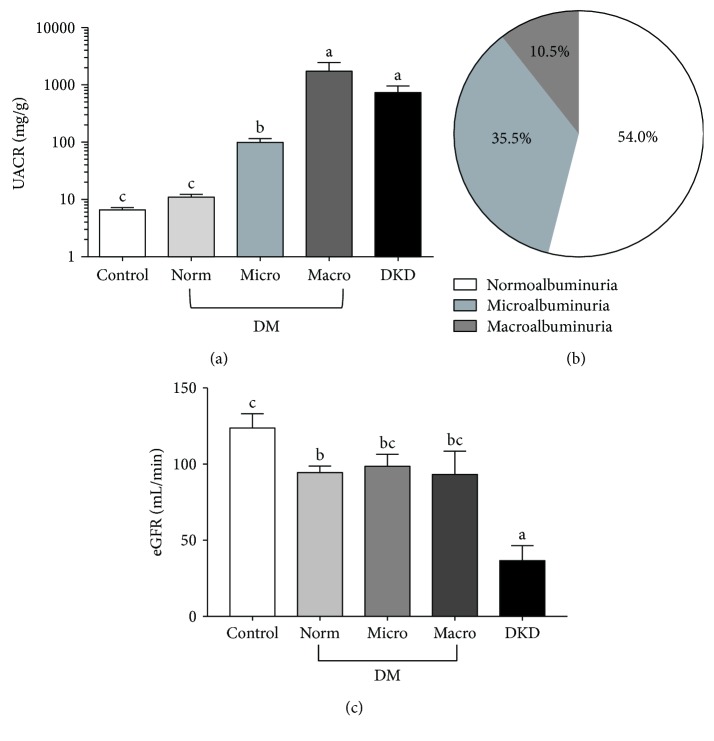
Biochemical assessment of diabetic kidney disease. (a) Urinary albumin-to-creatinine ratios (UACRs) among nondiabetic controls, diabetics with no known kidney disease (DM), and diabetics with diagnosed diabetic kidney disease (DKD). Diabetic patients were divided into three groups; normo-, micro-, and macroalbuminuria based on their UACR levels. Data are represented as mean ± standard error about the mean (SEM). Labels without a common letter indicate a significant difference between the groups (*p* < 0.05). (b) The proportion of diabetic patients with no known kidney disease with UACRs in the normo- (UACR ≤ 30 mg/g), micro- (30 ≤ UACR ≤ 300 mg/g), and macroalbuminuria (300 mg/g ≤ UACR) ranges. (c) Average estimated glomerular filtration rates (eGFR) among nondiabetic controls, diabetics with no known kidney disease (DM), and diabetics with diagnosed DKD. Members of the DM group are subdivided into normo-, micro-, and macroalbuminuria groups, as in (a). Data are represented as mean ± SEM. Labels without a common letter indicate a significant difference between the groups (*p* < 0.05).

**Figure 2:**
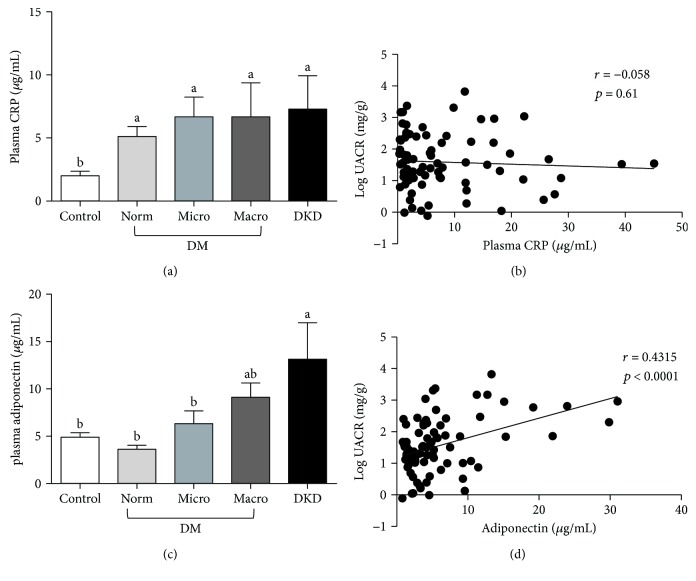
Plasma levels for C-reactive protein (CRP) (a) and adiponectin (c) in diabetic African American men and age-matched nondiabetic controls and diabetics with known DKD. The correlation between plasma CRP (b) or adiponectin (d) and log-transformed UACR in diabetic and DKD groups. Data are represented as mean ± SEM. Labeled means without a common letter are significantly different (*p* < 0.05).

**Figure 3:**
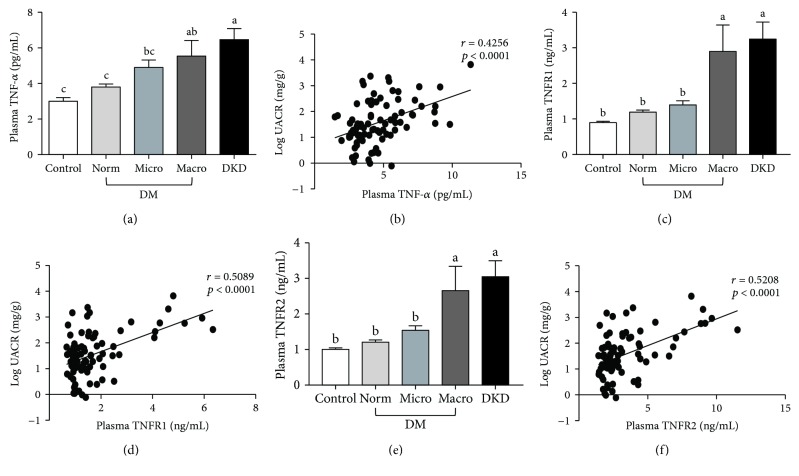
Plasma TNF-*α* (a), TNFR1 (c), and TNFR2 (e) levels in diabetic African American men, age-matched nondiabetic controls, and diabetics with diagnosed DKD. The correlation between plasma TNF-*α* (b), TNFR1 (d), and TNFR2 (f) and log-transformed UACR in diabetic and DKD groups. Data are represented as mean ± SEM. Labeled means without a common letter are significantly different (*p* < 0.05).

**Figure 4:**
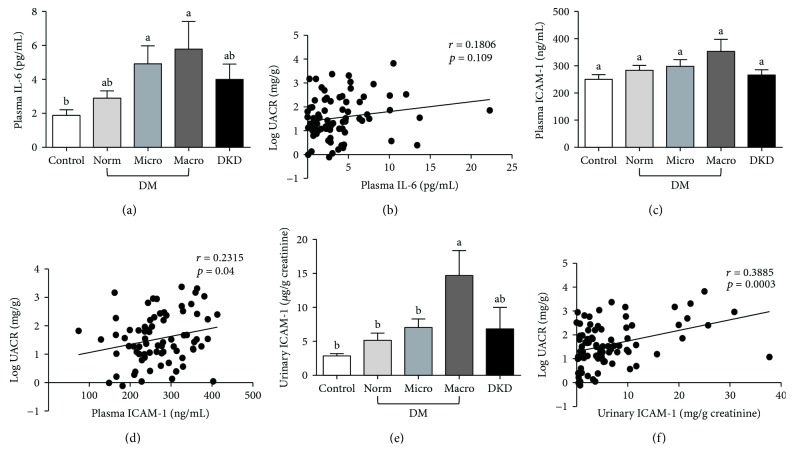
Plasma IL-6 (a), ICAM-1 (c), and urinary ICAM-1 (e) in diabetic African American men, age-matched nondiabetic controls, and diabetic patients with diagnosed DKD. Diabetic patients were classified into three groups: normo-, micro-, and macroalbuminuria, based on the UACR. The correlation between plasma IL-6 (b), ICAM-1 (d), or urinary ICAM-1 (f) and log-transformed UACR in diabetic and DKD groups. Data are represented as mean ± SEM. Labeled means without a common letter are significantly different (*p* < 0.05).

To better understand the relationship between inflammation and DKD in African American men, diabetic and nondiabetic participants were recruited from Greensboro, NC, and the surrounding area. Participants were divided into three groups based on clinical diagnosis of diabetes and diabetic kidney disease (DKD). The three groups consisted of (i) nondiabetic controls (*n* = 81), (ii) diabetic patients with no known kidney disease (*n* = 87), and (iii) diabetic patients with diagnosed DKD (*n* = 20). The clinical characteristics of the subjects are summarized in Table 1. There were no significant differences among the three groups with regard to age, BMI, or blood pressure. However, on average, diabetic patients either with or without diagnosed DKD had greater waist circumferences than nondiabetic controls. To determine the proportion of subjects within each group that exhibited impaired kidney function, we first measured the urinary albumin-to-creatinine ratio (UACR) for the participants in each group (Figure 1). The UACR was then used to further subcategorize patients in the diabetic group into those with normoalbuminuria, microalbuminuria, and macroalbuminuria (Figures 1(a) and 1(b)). We also calculated the estimated glomerular filtration rate (eGFR) for each group using the CKD-EPI creatinine-cystatin C equation (Figure 1(c)) [37]. However, no significant differences in eGFR were observed among diabetic patients with normo-, micro-, or macroalbuminuria, suggesting that UACR may be more reliable for predicting early onset of diabetic kidney injury in this subset of patients. We then compared the levels of seven protein markers of inflammation among members of each group. Specifically, we examined two proteins, adiponectin and CRP, associated with system-wide inflammation (Figure 2). The other five markers, TNF-*α*, TNFR1, TNFR2, IL-6, and ICAM-1, are known to be involved in renal inflammation (Figures 3 and 4).

### 3.2. Markers of System-Wide Inflammation

Consistent with previous reports that plasma CRP is a predictor of diabetes [39], plasma CRP levels in our subjects were elevated in diabetic patients compared to nondiabetic controls (Figure 2(a)). However, CRP levels did not appear to correlate with the extent of diabetic kidney injury, as determined by UACR (Figure 2(b)). Unlike CRP, plasma levels of the anti-inflammatory protein, adiponectin, did appear to vary with diabetic kidney injury status (Figure 2(c)). For instance, while nondiabetic controls and diabetics with normo- and microalbuminuria had comparable plasma adiponectin levels, the plasma adiponectin levels were between 2 and 3 times higher in patients with macroalbuminuria and between 2.3 to 4 times higher in patients with DKD (Figure 2(c)). Importantly, adiponectin levels positively correlated with log-transformed UACR (*r* = 0.4315; *p* ≤ 0.001) (Figure 2(d)). Also, consistent with previous studies, adiponectin exhibited a negative correlation with respect to both BMI and WC in our cohort (Table 2).

### 3.3. Markers Involved in Renal Inflammation

Next, we asked if similar relationships existed between kidney injury status and protein markers associated with renal inflammation. While there was no significant difference in plasma TNF-*α* levels in diabetic patients with normoalbuminuria compared to nondiabetic controls, the levels of TNF-*α* increased steadily with progression from normo- (3.8 ± 0.173 pg/mL) to micro- (4.901 ± 0.425 pg/mL), and macroalbuminuria (5.534 ± 0.89 pg/mL) (Figure 3(a)). As a consequence, there was a significant positive correlation between plasma TNF-*α* and log-transformed UACR from diabetic patients (*r* = 0.4256; *p* < 0.0001) (Figure 3(b)). Likewise, relative to nondiabetic controls, there was a strong correlation between log-transformed UACR and both plasma TNFR1 (*r* = 0.5089, *p* < 0.0001) and TNFR2 (*r* = 0.5208, *p* < 0.0001) (Figures 3(c)–3(f)). The correlation between TNF receptors and kidney injury was further corroborated by correlation analysis with two other kidney injury markers, namely, urinary KIM-1 and NGAL (Table 3). Both TNFR1 and TNFR2 have moderate but very evident correlations with these two kidney injury markers (Table 3). Together, these data suggest that inflammatory processes mediated by the TNF-*α*/TNFR/NF-*κ*B signaling axis may play an important role in the progression of DKD in our patient population.

To explore this hypothesis further, we examined the levels of two other inflammatory factors whose expression is regulated by TNF-*α*/TNFR/NF-*κ*B signaling, namely, IL-6 and ICAM-1 (Figure 4) [40, 41]. These experiments revealed a significant increase in plasma levels of IL-6 among diabetic patients with micro- and macroalbuminuria (Figure 4(a)). However, unlike TNF-*α*, TNFR1, and TNFR2, plasma IL-6 levels were only weakly correlated with the log UACR (*r* = 0.1806) (Figure 4(b)). Moreover, the correlation was not significant (*p* = 0.109). Likewise, though modest increases in plasma ICAM-1 levels were observed with increasing severity of kidney injury, there were no significant differences between the levels found in diabetic patients when compared to those found in nondiabetic controls (Figures 4(c) and 4(d)). In contrast, the levels of urinary ICAM-1 increased steadily among diabetic patients and were significantly higher in diabetic patients with macroalbuminuria compared to nondiabetic controls (*p* = 0.0001) (Figures 4(e) and 4(f)). Furthermore, the fold difference between nondiabetic controls and diabetic patients with macroalbuminuria was much higher for urinary ICAM-1 (~5.8-fold) than for plasma ICAM-1 (~1.4-fold; Figures 4(c) and 4(e)). Likewise, urinary ICAM-1 levels were approximately 3-times higher in the macroalbuminuric group (14.69 ± 3.66 *μ*g/g creatinine) than in the normoalbuminuric group (5.14 ± 1.07 *μ*g/g creatinine) (Figure 4(e)). Importantly, the urinary levels of ICAM-1 positively correlated with the severity of kidney injury (Figure 4(f)). Interestingly, the correlation analysis also indicated a stronger association between log-transformed UACR and urinary ICAM-1 than plasma ICAM-1 (*r* = 0.3885, *p* = 0.0003, for urinary ICAM-1 vs. *r* = 0.2315, *p* = 0.04, for plasma ICAM-1). This may suggest that urinary ICAM-1 is a more sensitive predictor of diabetic kidney injury than plasma ICAM-1 in our population.

**Figure 5:**
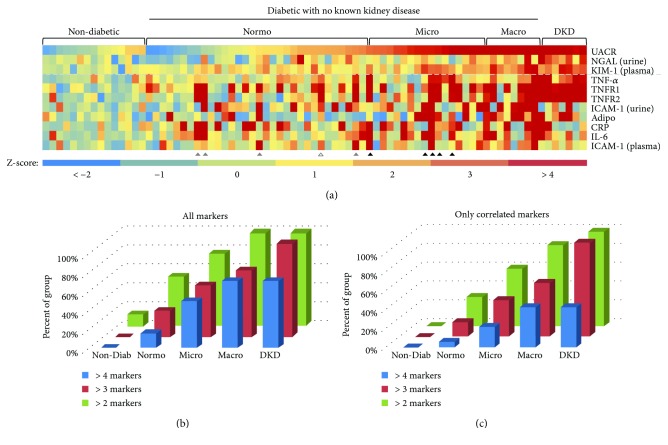
(a) Heat map showing the relative levels of 11 markers from subjects from different subgroups based on albuminurea. Each column represents one subject from our study, with the level of each of the 11 markers indicated by a Z-score relative to the mean of the nondiabetic controls for that marker. The relative levels of 3 kidney injury markers (i.e., log-transformed UACR, urinary NGAL and plasma KIM-1) are shown above the dashed line while the relative levels of 8 inflammation markers are shown below the dashed line. Higher Z-scores are represented by warmer colors while lower Z-scores are represented by cooler colors. Individuals who exhibited elevated Z-scores for at least 3 of the correlated inflammation markers, despite being in the normoalbinurea group (grey triangles) or the microalbinurea group (black triangles) based on UACR, are indicated. (b) Percentage of subjects from each group with between 2 and 4 inflammation markers whose levels were at least 2 standard deviations above the mean of the nondiabetic group, among all markers tested. (c) Percentage of subjects from each group with between 2 and 4 inflammation markers whose levels were at least 2 standard deviations above the mean of the nondiabetic group, among correlated markers only (i.e., plasma adiponectin, TNF-*α*, TNFR1, TNFR2, ICAM-1, and urinary ICAM-1).

Since several of the inflammation markers that we examined correlated with the severity of kidney injury in diabetic patients, we were curious whether individuals within each group exhibited elevated levels for multiple markers. Therefore, we constructed a heat map that captures an apparent “inflammation profile” for each individual in the study (Figure 5(a)). During these analyses, we also included data for the kidney-specific markers, plasma KIM-1 and urinary NGAL, that we recently showed to correlate with the extent of DKD in this population [38]. These analyses suggest that the number of individuals with elevated levels of multiple inflammation markers increased with the severity of kidney injury, as measured by UACR (Figures 5(b) and 5(c)). This trend was observed if we examined all inflammation markers (Figure 5(b)) or only those markers that correlated with log-transformed UACR in the ensemble analyses (i.e., adiponectin, TNF-*α*, TNFR1, TNFR2, and urinary ICAM-1) (Figure 5(c)). Importantly, chi-square analysis suggests that the observed trends are statistically significant in both cases. Interestingly, several diabetic patients with microalbuminuria, and even a few with normoalbuminuria, exhibited elevated levels of three or more inflammation markers (Figure 5(a), triangles). In most cases, these individuals also exhibited elevated levels of at least one kidney-specific marker. In the future, it will be interesting to see if these individuals progress more rapidly to macroalbuminuria and later stages of kidney injury than those patients with elevated levels of only one (or fewer) inflammation markers.

## 4. Discussion

In the United States, diabetes and complications of diabetes are more prevalent in minority ethnic populations, such as African Americans, Native Americans, and Mexican Americans, yet Caucasians are the most widely studied ethnic group [42, 43]. Studies focused on African American men are particularly rare. The data drawn from other races or gender groups cannot always be generalized to African American men due, in part, to genetic, gender, and cultural factors. In this study, we sought to identify biomarkers of inflammation that were correlated with diabetic kidney injury in African American men, a subpopulation that is understudied. To this end, we partnered with the Cone Health Community Health and Wellness Center (Greensboro, NC), a community clinic that serves predominantly uninsured and underinsured patients. A substantial proportion of diabetic patients in the current study had significantly higher waist circumferences when compared to the nondiabetic controls. This may suggest that waist circumference is a better predictor for T2D than BMI in this population. In support of this notion, Kahn et al. identified a correlation between waist circumference and risk for T2D in other ethnic groups [44].

Analysis of plasma and urine samples showed significant correlations between progression of kidney injury and several inflammatory markers. Two of the markers analyzed in our study (adiponectin and CRP) are indicative of system-wide inflammation, while the other five (TNF-*α*, TNFR1, TNFR2, IL-6, and ICAM-1) have been shown to play a role in renal inflammation. Existing clinical data indicate that diabetes, as a whole, is strongly associated with elevated levels of IL-6, TNF-*α*, CRP, and adiponectin in Mexican Americans [45]. Likewise, elevated plasma levels of IL-6 could predict the development of T2D in women [46]. Our data show that five inflammation markers (TNF-*α*, TNFR1, TNFR2, ICAM-1, and adiponectin) also correlated with the extent of kidney injury in African American men, as determined by the UACR. On the other hand, the plasma levels of IL-6 and CRP were not significantly correlated with kidney injury in our population.

In the current study, plasma TNF-*α* levels were significantly higher in diabetic subjects than in nondiabetic controls. Moreover, TNF-*α* levels also positively correlated with kidney injury, as determined by UACR. Interestingly, the levels of the TNF receptors (TNFR1 and TNFR2) also strongly correlated with the UACR as well as with the urinary levels of two proteomic markers of kidney injury, KIM-1 and NGAL. However, TNF-*α* did not show a significant correlation with KIM-1 or NGAL. Taken together, these results corroborate the role for the TNF-*α*/TNFR/NF-*κ*B signaling axis in the progression of diabetes and diabetic kidney injury. As a key regulator of apoptotic signaling pathways, TNF-*α* is cytotoxic to renal cells and is thus able to induce direct renal injury [47]. It prompts the local generation of reactive oxygen species, which results in alterations in the barrier function of the glomerular capillary wall leading to enhanced albumin permeability [48]. Previous studies have also identified TNF-*α* as a marker for kidney injury in other ethnic groups. For instance, a study of Japanese patients with microalbuminuria showed higher serum TNF-*α* levels than those without microalbuminuria did [21]. Likewise, previous studies found that albuminuria is associated with elevated serum TNF-*α* in Spanish patients with T2D [49]. We also observed an increase in plasma IL-6 in patients with micro- and macroalbuminuria when compared to nondiabetic controls. However, plasma IL-6 levels did not significantly increase with the progression from micro- to macroalbuminuria. Since kidneys may play a role in the clearance of inflammatory cytokines, such as IL-6 and TNF-*α* [50], it is possible that the elevated plasma levels of these two factors might indicate impaired kidney function. Indeed, we observed significant differences in eGFR between nondiabetic controls, diabetics without kidney disease, and patients with diagnosed DKD (interestingly, however, no significant differences were observed between participants within the diabetic subgroups based on albuminuria status, suggesting that eGFR may not be a reliable predictor of kidney injury in this patient subpopulation). IL-6 has been shown to have context-dependent pro- and anti-inflammatory properties [51]. The renal effects of IL-6 include increased endothelial permeability and growth of renal mesangial cells [52]. Consistent with this role, plasma IL-6 was shown to have a positive correlation with diabetic kidney injury in a Japanese population [21].

The current study also demonstrated a positive correlation between UACR and urinary ICAM-1, suggesting that ICAM-1 could serve as an indicator of the progression of diabetic kidney injury in African American men. Furthermore, discrepancies between the fold change in plasma and urinary levels of ICAM-1 suggest that urinary ICAM-1 is more sensitive as a predictor of diabetic kidney injury than plasma ICAM-1. ICAM-1 promotes inflammation by enhancing leukocyte infiltration and adherence [13]. ICAM-1 levels are elevated in the serum of patients with cardiovascular disease, autoimmune disorders, and cancer [53]. Meanwhile, urinary ICAM-1 levels increased in patients with kidney diseases [54]. In lupus nephritis patients, levels of urinary ICAM-1 were significantly higher in patients with advanced histological changes than in other groups [55]. It was found that urinary ICAM-1-to-creatinine ratios in patients with T2D and microalbuminuria were much higher than those in nondiabetic controls in a Chinese population [56].

Using correlation analysis, we observed a positive correlation between UACR and adiponectin among the African American men in our study. Produced almost exclusively by adipocytes, adiponectin exerts anti-inflammatory actions [57]. In previous studies, adiponectin levels were inversely associated with the risk of T2D [58]. Mixed results on the correlation between plasma adiponectin and DKD have been reported. For instance, severe proteinuria was associated with decreased plasma adiponectin in a Turkish hospital [59]. In contrast, serum adiponectin levels were found to be significantly higher in patients with macroalbuminuria compared to those with normoalbuminuria in a Japanese study [60]. In the current study, the plasma adiponectin levels in the macroalbuminuria group were significantly higher than all the other groups. CRP is the principal downstream mediator of the acute phase response [61]. It is also considered a diabetic risk predictor [46]. Expression of CRP is regulated mainly at the transcriptional level, with IL-6 being the principal inducer of the gene during the acute phase. Plasma CRP levels may have some race/ethnic-specific characteristics. For instance, compared to women in other ethnic groups, African American women had the highest median CRP concentrations [62]. Plasma CRP increased with increased excretion of albumin in a Netherland population with type I diabetic mellitus [63] and an Iranian population [28], while it was not associated with albuminuria in multivariable-adjusted analyses in African Americans [64]. In our study, the increased plasma CRP was seen in diabetic subjects, but did not correlate with the albuminuria status. This suggests that the progression of diabetic kidney injury is unlikely to be related to CRP in this population.

Finally, our data suggest that integrated analysis of a panel of inflammation markers can provide unique insights into a disease state that may not be apparent from analysis of a single marker. For instance, we observed an increase in the number of inflammatory markers with increasing severity of DKD (Figure 5). In many cases, the elevated markers were found to cluster among proteins within the same signaling pathway. For instance, several individuals who exhibited elevated levels of TNF-*α* also had increased levels of the TNF receptors, TNFR1 and TNFR2, as well as proteins whose expression is regulated by the TNF-*α*/TNFR/NF-*κ*B signaling axis, such as ICAM-1 and IL-6. Consistently, several studies have recently highlighted the nephroprotective effects of pharmacological agents that attenuate inflammatory processes via inhibition of the NF-*κ*B signaling pathway [65, 66]. Therefore, this type of integrated analysis may offer valuable insights into the cellular mechanisms driving disease progression in these individuals. In conclusion, findings from the current study corroborate the notion that inflammation is an important factor in the pathogenesis of DKD in African American men.

## Figures and Tables

**Table 1:** Clinical characteristic of nondiabetic and diabetic subjects.

	Nondiabetic controls (*n* = 81)	Diabetics (*n* = 87)	Diabetics with diagnosed kidney disease (*n* = 20)
Age (years)	45.4 ± 1.48^a^	49.6 ± 1.20^a^	48.5 ± 2.08^a^
Body mass index (kg/m^2^)	30.5 ± 0.89^a^	32.6 ± 0.87^a^	32.1 ± 2.3^a^
Waist circumstance (cm)	101.0 ± 2.15^b^	112.7 ± 2.06^a^	112.7 ± 5.34^ab^
Blood pressure (mmHg)			
Systolic	135.9 ± 2.37^a^	136.0 ± 2.32^a^	140.4 ± 4.63^a^
Diastolic	87.1 ± 1.49^a^	85.6 ± 1.39^a^	87.9 ± 2.81^a^
Glycated hemoglobin (A1c)	N/A	9.47 ± 0.38^a^	8.54 ± 1.017^a^

Data are represented as mean ± SEM. Labeled means without a common letter differ (*p* < 0.05).

**Table 2:** Correlation coefficient between inflammation markers and clinical characteristics in diabetic African American men.

	CRP	Adiponectin	TNF-*α*	TNFR1	TNFR2	IL-6	Plasma ICAM-1	Urinary ICAM-1
BMI	0.38 (0.0006)	−0.34 (0.0018)	−0.056 (0.61)	−0.054 (0.62)	−0.010 (0.36)	0.35 (0.0017)	0.061 (0.59)	0.037 (0.74)
WC	0.34 (0.0031)	−0.44 (<0.0001)	−0.010 (0.36)	−0.13 (0.24)	−0.089 (0.44)	0.25 (0.03)	0.095 (0.42)	−0.018 (0.88)
SBP	0.042 (0.71)	0.055 (0.62)	0.17 (0.13)	0.11 (0.29)	0.093 (0.40)	0.091 (0.43)	0.12 (0.28)	0.22, (0.052)
DBP	0.061 (0.59)	−0.042 (0.70)	0.025 (0.82)	0.18 (0.089)	0.14 (0.19)	0.0049 (0.97)	0.14 (0.20)	0.19 (0.082)

BMI: body mass index; WC: waist circumference; SBP: systolic blood pressure; DSP: diastolic blood pressure. Data are *r* values based on Pearson's coefficient correlations; *p* values are in parentheses.

**Table 3:** Correlation coefficient between TNF-related markers and KIM-1 and NGAL in diabetic African American men.

	TNF-*α*	TNFR1	TNFR2
Log uKIM-1	0.11 (0.34)	0.34 (0.0023)	0.33 (0.0047)
Log pKIM-1	0.53 (<0.0001)	0.63 (<0.0001)	0.61 (<0.0001)
Log uNGAL	0.21 (0.09)	0.46 (<0.0001)	0.46 (<0.0001)

Data are *r* values based on Pearson's coefficient correlations; *p* values are in parentheses.

## Data Availability

The data used to support the findings of this study are included within the article.
